# Meso- or xeromorphic? Foliar characters of Asteraceae in a xeric scrub of Mexico

**DOI:** 10.1186/s40529-017-0166-x

**Published:** 2017-02-23

**Authors:** Patricia Rivera, José Luis Villaseñor, Teresa Terrazas

**Affiliations:** 1 Departamento de Botánica, Instituto de Biología, Universidad Nacional Autónoma de México, Apartado Postal 70-367, 04510 Mexico City, Mexico; 2 Coordinación del Posgrado en Ciencias Biológicas, Circuito de Posgrados Ciudad Universitaria, Coyoacán, 04510 Mexico City, Mexico

**Keywords:** Astereae, Eupatorieae, Heliantheae, Foliar anatomy, Poor soils, Water stress

## Abstract

**Background:**

The anatomical traits associated with water deficit are also observed in plants growing in poor soils. The species may resist water deficit through three main strategies: escape, avoid or tolerate. The Pedregal de San Ángel Ecological Reserve (REPSA), Mexico, is an environment with low nutrient soil and low water availability. It is set on the basalt formation derived from the Xitle volcano eruption. The main vegetation type is characterized as xerophytic shrub. Thus we expect that species growing in this community will show leaf xeromorphic traits and may have any of the three response strategies. We analyzed the foliar anatomy of 52 species of the Asteraceae family at the REPSA because it is the most abundant angiosperm family in the site, showing a wide variety of growth forms and anatomical variation.

**Results:**

The foliar anatomies of the studied Asteraceae were highly variable as well as their quantitative traits as revealed by principal component analysis. This agrees with previous studies that found great anatomical variation within the family. Leaves have multiple layered palisade parenchyma and parenchyma bundle sheaths and could not be categorized as xeromorphic because they possess mesomorphic leaf features as simple lamina, single-layered epidermis, and soft large-size glabrous leaves with high specific leaf area.

**Conclusions:**

The combination of mesomorphic and few xeromorphic foliar traits with other characters at the genus and tribal level probably has been essential in Asteraceae to colonize various environments, including those with low water and nutrient availability.

**Electronic supplementary material:**

The online version of this article (doi:10.1186/s40529-017-0166-x) contains supplementary material, which is available to authorized users.

## Background

Plants growing under xeric conditions can have a set of anatomical, physiological or phenological adaptations allowing them to escape, avoid or tolerate water stress (Santos and Ochoa 1990; Fahn and Cutler 1992; Dickison 2000; Valladares et al. 2004). Drought-escaping species generally are annual or biannual herbs that develop their life-cycle in short time periods when conditions are favorable (Kramer 1983; Santos and Ochoa 1990; Fahn and Cutler 1992; Valladares et al. 2004) or they usually possess perennation structures, such as bulbs, rhizomes or runners that remain latent until conditions are favorable again. Plants showing the avoidance-of-stress strategy possess some features enabling them to minimize or compensate for water loss or to increase water uptake and avoid desiccation. Indicator traits of the drought avoidance mechanism include deep roots, small or strongly lobulated leaves, mesophyll cells smaller in size, thick cell-walls and cuticles, multiseriate epidermis, strongly developed palisade parenchyma, stomata sunken or in crypts and abundant trichomes (Esau 1976; Kramer 1983; Santos and Ochoa 1990; Gibson 1996; Dickison 2000; Valladares et al. 2004; Fang and Xiong 2015). Drought tolerating plants can sustain a certain level of physiological activities under stress conditions. Tolerating plants show tissues resistant to dehydration and characters such as stress-induced leaf abscission, succulent leaves, lignified cell-walls, mucilage accumulation and the occurrence of buliform cells (Kramer 1983; Santos and Ochoa 1990; Dickison 2000; Valladares et al. 2004; Fang and Xiong 2015).

Traits of stress-avoiding and stress-tolerating plants have also been related to soil nutrient deficit (Dickison 2000; Fonseca et al. 2000; Wright et al. 2002). In fact, soil nutrient availability explains better the distribution of foliar characters in plants around the world than mean annual temperature, mean annual precipitation and irradiance together (Ordoñez et al. 2009). It has been hypothesized that xeromorphism has evolved from the adaptation of mesomorphic plants to low nutrient soil (Fahn and Cutler 1992) or to both, the lack of water and poor soils. These two conditions are found at the Pedregal de San Ángel Ecological Reserve in the Basin of Mexico.

**Fig. 1:**
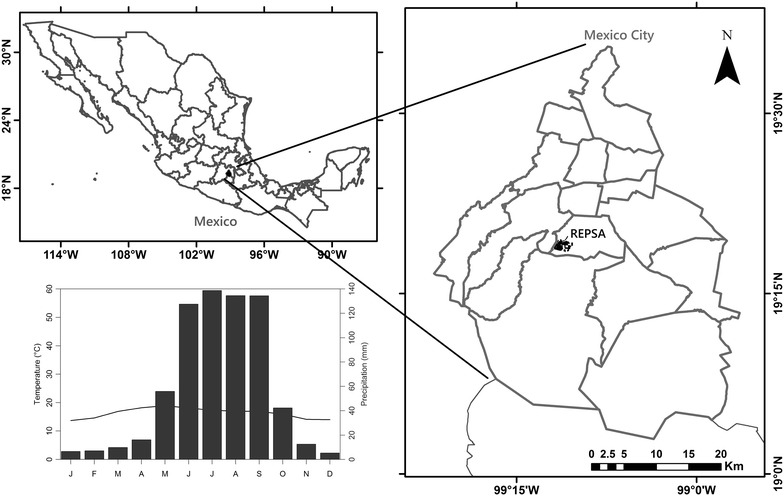
Location and some characteristics of the study site, the Pedregal de San Ángel Ecological Reserve-REPSA. **a** Geographic location of the REPSA in Mexico. The REPSA is marked as the *black shaded area* in Mexico City. **b** Climograph of the REPSA

The Pedregal de San Ángel Ecological Reserve (REPSA) is a protected area for the vegetation at the southern edge of the Mexico basin (Fig. 1a). The REPSA is located in the southwest region of the Mexico City basin (19°18′21″ to 19°20′11″N and 99°10′15″ to 99°12′4″W), at an elevation interval of 2292–2365 m, and it encompasses an area of 237.3 ha (Fig. 1a). The vegetation type has been characterized as xerophilous scrub (Rzedowski 2006). This ecosystem is on a set of basaltic formations produced by the solidification of lava flow during the eruption of the Xitle volcano about 1670 years ago (Siebe 2000). Although mean annual precipitation is 883 mm, the bedrock does not allow water retention and the soil is shallow. So aridity in this site is more edaphic than climatic. It is a unique system in the world because it is within the biogeographic province of the Transmexican Volcanic Belt (Morrone 2006) and the flora composition has Neotropical and Nearctic affinities (Rzedowski 1998; Rzedowski and Rzedowski 2005). The vegetation surrounding the REPSA includes various types of forests, including oak forest, fir forest, and cloud forest (Rzedowski and Rzedowski 2005; Lot and Cano-Santana 2009), which is consistent with the vegetation type expected given the elevation (over 2240 masl), the mean annual rainfall (770–1200 mm), and the mean temperature (11–15 °C) of the site (Fig. 1b). Therefore, it is assumed that species hat colonized after the eruption came mainly from the surrounding forests; however, the current vegetation type is xerophilous scrub (Castillo-Argüero et al. 2007; Lot and Cano-Santana 2009). At the REPSA, the Asteraceae family shows the highest number of species compared to the 73 other vascular plant families found there (Castillo-Argüero et al. 2004, 2007; Lot and Cano-Santana 2009). Céspedes (2010) reported 93 species belonging to 51 genera classified in 13 tribes of Asteraceae in this site. Moreover, the morphological diversity of the family is well represented, as there is a wide variety of growth forms, polyploid species and species with high chromosomic numbers (Soto-Trejo et al. 2011).

The foliar anatomy of Asteraceae has been studied mainly in species with economical value (Ragonese 1988; Ferreira et al. 2002; Freire et al. 2005, 2007; Milán et al. 2006). The leaves are usually dorsiventral, hypo- or amphistomatic and have anomocytic stomata. However, it is difficult to assert generalizations regarding the foliar anatomy of Asteraceae because there is so much variation in characters such as stomata distribution, trichome density and type, hypodermis development, presence of secretory structures and parenchymatic vascular bundles sheaths (Anderson and Creech 1975; Metcalfe and Chalk 1979; Freire et al. 2005). The usefulness of foliar characters for taxonomic classification at the genus or species level (Ferreira and De Oliveira 1989; Castro et al. 1997; Luque et al. 1999; Delbón et al. 2007; Adedeji and Jewoola 2008) or for phylogenetic and ecological studies (Boeger and Wisniewski 2003; Horn et al. 2009) has been overlooked because most studies have focused exclusively on examining the epidermal surface (Ferreira et al. 2002; Freire et al. 2005, 2007; Adedeji and Jewoola 2008; Gil et al. 2012; Redonda-Martínez et al. 2016) and ecological or leaf evolution traits are missing for Asteraceae as they have been generated for other plant families (Luckow 2002; Schmerler et al. 2012; Brodribb et al. 2013).

We hypothesize that Asteraceae species at this ecological protected area (REPSA) will show anatomical traits for avoiding or tolerating stress, the aim of this study was to characterize the foliar anatomy of a sample of 52 species belonging to 41 genera and 13 tribes of Asteraceae living in the xeric environment of this reserve.

## Methods

The present study was conducted in the REPSA, within the central campus of the National Autonomous University of Mexico (UNAM, Fig. 1a). Samples were collected during two rainy seasons from August 2008 to December 2009. Three to six fully developed leaves showing no evidence of injury were removed from three individuals of each selected species. All the leaves were photographed with a digital camera. Leaf area was determined using an image analyzer according to the procedure described by Garnier et al. (2001). After being photographed, two to three the leaf laminas per species were dried in oven at 60 °C for at least 48 h to constant weight and then weighed on an analytical balance to determine the dry mass of the leaf. Specific leaf area (SLA) was calculated as the ratio between the leaf area and leaf dry mass [SLA = leaf area (cm^2^)/dry leaf mass (g)]. The remaining leaves (1–4 per species) were fixed with a formaldehyde-glacial acetic acid-ethyl alcohol solution (Ruzin 1999). They were then rinsed with tap water to remove fixative residues and stored in a glycerin-ethyl-alcohol-water solution (1:1:1) until sectioning. Voucher specimens information is given in Additional file 1.

Portions of the middle region of the leaf, including the intercostal area from the midvein to the margin were cut, rinsed and dehydrated in increasing concentrations of tert-butanol (10–100%) with a Leica automatic tissue processor (TP1020) remaining for 24 h in each concentration. The tissues were embedded in paraffin (melting point: 56 °C) and transverse and paradermal sections 10–12 µm in thickness were cut with a Leica rotatory microtome (RM2125). The resulting sections were stained with safranin-fast green (Johansen 1940) and mounted on synthetic resin. In the paradermal sections, the cuticle, guard cell length and epidermal cell shapes were examined. In the transverse sections, the thickness of the following features was measured: the total leaf, mesophyll, palisade parenchyma, spongy parenchyma, cuticle, and height of the abaxial and adaxial epidermis cells. The measurements were obtained through an Olympus microscope BX-51 attached to an image analyzer (Image Pro-plus version 6.1, Media Cybernetics 2006). For each variable, 25 measures were recorded. Other features, such as the number of palisade parenchyma layers or the spongy parenchyma type (open or compact), were also determined. Laminar size classification followed Webb (1959), detecting 6 categories: nanophyll (0.25–2.25 cm^2^), microphyll (2.25–20.25 cm^2^), notophyll (20.25–45 cm^2^), mesophyll (45–182.25 cm^2^), macrophyll (182.25–1640.25 cm^2^) and megaphylly (>1640.25 cm^2^). Sclerophylly index (SI) was calculated following Boeger and Wisniewski (2003) where SI = leaf dry mass (g)/2 leaf area (cm^2^), considering SI > 0.6 as sclerophylly and SI < 0.6 as mesophylly). Stomatal pore area index (SPI) was calculated following Tian et al. (2016) where SPI = stomatal density*stomatal length*10^−4^. Leaf lamina and midvein descriptions followed Dickison (2000) and tribe classification followed Funk et al. (2009).

Characters were square root and log10-transformed prior to statistical analyses. Pearson and Sperman correlations were calculated between pairs of leaf traits. In addition we produce a principal component analysis (PCA) to evaluate which leaf traits explained the variation in this site. Analyses were performed with R (R Core Team 2015) and SAS (Statistical Analysis System software version 9, SAS Institute 2002).

## Results

### Leaf morphology

The leaves were simple in all the species. However, 29% of species, including most of the studied species of the Tageteae tribe, were deeply lobed. Based on the laminar size, most species were classified in four categories: microphyll (29% of the species), mesophyll (29%), notophyll (23%) and macrophyll (13%). Only two species (*Montanoa grandiflora* and *Tithonia tubiformis*) were classified as megaphyll and one species (*Tagetes micrantha*) as nanophyll leaves. Laminar shape was variable, from ovate, obovate to elliptic. Toothed or untoothed leaf margins were observed. Serrate margins were common and crenate margins were present in some species. Leaf morphological characters by species are given in Additional file 2.

### Epidermis

**Fig. 2:**
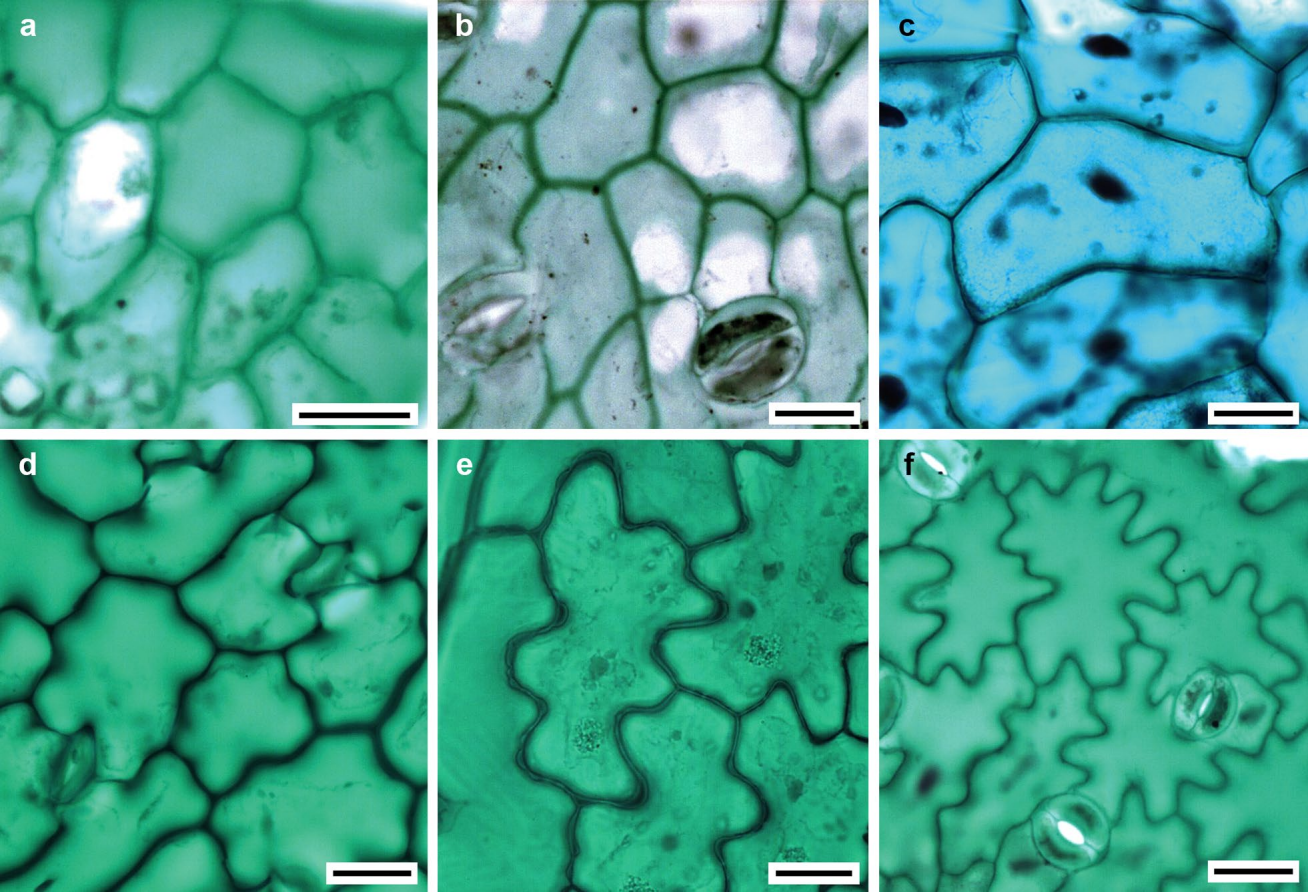
Epidermal cells forms of Asteraceae. **a** *Conyza canadensis* (Tribe Astereae). **b** *Barckleyanthus salicifolius* (Senecioneae). **c** *Pittocaulon praecox* (Senecioneae). **d** *Pectis prostrata* (Tageteae). **e** *Ageratina adenophora* (Eupatorieae). **f** *Lactuca serriola* (Cichorieae). *Bar* is 20 µm

On the surface, the abaxial and adaxial epidermises were glabrous in 95% of the examined species. The remaining 5% were glandular, tector or pluricellular uniseriate or possessed multiseriate trichomes. Trichomes could be found on one or both leaf surfaces, but they were more frequent on the abaxial surface. The epidermal cell shape of both surfaces was elongated polygonal or isodiametrical polygonal, with straight (Fig. 2a–c) or wavy anticlinal walls (Fig. 2d–f). Both amphistomatic and hypostomatic leaves were observed. When amphistomatic, the stomata were more abundant on the abaxial surface. The stomata were anomocytic and rarely anisocytic. The average stomatal density was 362 stomata/mm^2^, and the average per tribe is given in Table 1.

**Table 1:** Leaf characters by tribe in Asteraceae of the xeric scrub at the REPSA, Mexico

Tribe	Anthemidae	Astereae	Bahieae	Carduae	Cichorieae	Coreopsidae	Eupatorieae	Gnaphalieae	Heliantheae	Millerieae	Nassauvieae	Senecioneae	Tageteae
No. species/character	2	5	2	1	3	5	12	2	10	2	1	3	4
Leaf size classification	Micro*	Noto (3), micro (2)	Micro	Macro	Macro (2), meso	Meso (2), micro (2), noto (1)	Noto (5), meso (4), micro (2), macro (1)	Micro	Meso (4), noto (2), mega (2), macro (1), micro (1)	Meso, noto	Meso	Macro (2), meso	micro (2), meso (1), nano (1)
Lobation	Lobed	Unlobed (4), lobed	Lobed	Lobed	Lobed (2), unlobed	Lobed	Unlobed	Unlobed	Unlobed (7), lobed	Unlobed	Unlobed	Unlobed (2), lobed	Unlobed (2), lobed
Margin type	Untoothed	Untoothed (3), toothed	Untoothed	Tooted	Toothed (2), untoothed	toothed (3), untoothed	Toothed (11spp), untoothed	Untoothed	Toothed (8) or untoothed	Toothed	Toothed	Toothed (2spp) or untoothed	Toothed (2spp) or untoothed
Tooth type	NA	Serrated (2 spp)	NA	Serrated	Eentate	Serrated (3)	Serrated (11)	NA	Serrated (6), crenated (2)	Serrated	Serrated	Serrated (1) or dentate (1)	Serrated (2)
Leaf type by the position of stomata	Amphi*	Amphi	Amphi	Amphi	Amphi (2), hypos	Amphi (3), hypos	Hypos (8), amphi	Amphi (1), hypos	Amphi (7), hypos	Amphi	Amphi	Hypos (2), amphi	Amphi
	Bifacial or equifacial	Bifacial	Bifacial or equifacial	Bifacial	Bifacial	Bifacial or equifacial	Bifacial	Bifacial	Bifacial	Bifacial	Bifacial	Bifacial	Bifacial or equifacial
Total lamina thickness, mean µm	102.8	217.3	326.7	413.1	182.8	246.4	148.5	126.8	200.5	197.07	231.7	238.2	274.9
Cuticle surface	Striated (1), smooth	Striated	Striated	Striated	Striated (2), smooth	Striated	Striated (11), smooth	Smooth	Striated (7), smooth	Smooth	Striated	Striated	Striated (3), smooth
Cuticle thickness, range µm	0.31–0.43	0.27–0.56	0.32–0.37	0.32	0.30–0.45	0.22–0.41	0.20–0.60	0.33–0.42	0.21–1.15	0.23–0.24	<0.1	0.36–0.46	0.19–0.44
Epidermal cell walls (surface view)	Straight to wavy	Straight to wavy	Wavy	Straight	Straight to wavy	Straight to wavy	Wavy	Wavy	Straight to wavy	Wavy	Wavy	Straight to wavy	Straight to wavy
Position of stomata regarding ordinary epidermal cells	Same level	Same level	Same level	Same level	Same level	Same level	Same level (10), higher	Same level	Same level (10), higher	Same level	Same level	Same level	Same level
Stomatal density (abaxial epidermis, mean stomata/mm^2^)	244	369	366	349	322	383	421	349	394	279	174	256	349
Guard cell length, mean µm	21.36	21.04	23.8	24.38	18.7	21.61	19.64	17.89	20.31	20.18	25.09	23.09	19.39
Palisade parenchyma thickness, mean µm	22.48	69.99	117.65	195.58	46.2	98.03	47.29	44.43	76.27	54.34	52.40	45.47	72.68
Spongy parenchyma thickness, mean µm	53.38	48.20	58.08	165.68	90.94	92.89	71.48	52.97	81.54	113.32	140.46	157.6	93.12
Stomatal pore area index (SPI) %	10.37	15.53	15.70	20.75	15.06	17.27	14.56	11.13	15.09	11.39	10.98	13.57	13.80

In parenthesis number of species for each tribe

* See text for complete names

**Fig. 3:**
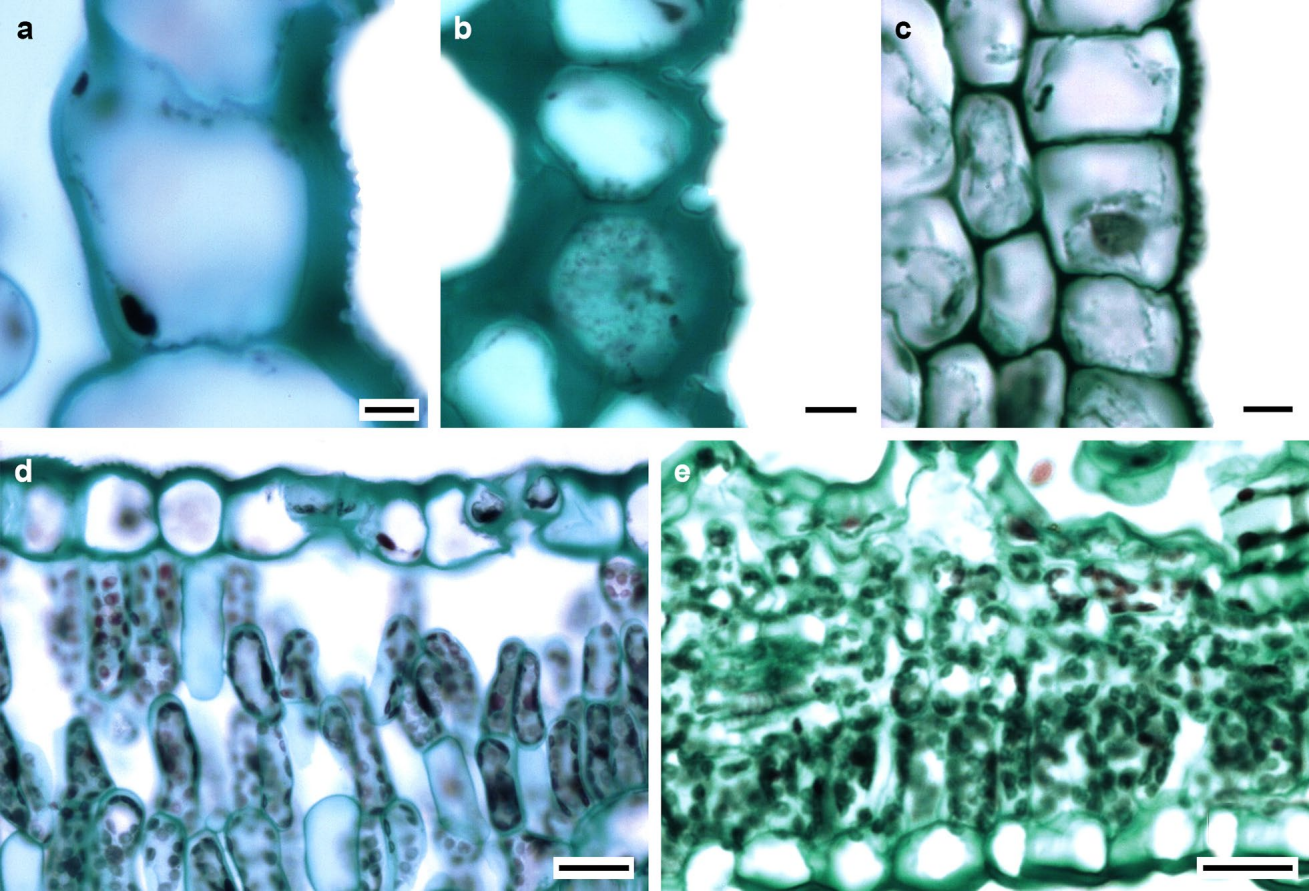
Cuticular striae and transverse view of stomata of some Asteraceae species. **a** *Baccharis salicifolia* (Tribe Astereae). **b** *Ambrosia psilostachya* (Heliantheae). **c** *Barckleyanthus salicifolius* (Senecioneae). **d** *Baccharis salicifolia* (Astereae). **e** *Brickellia secundiflora* (Eupatorieae). *Bar* is 5 µm in **a**–**c**, 20 µm in **d**, **e**

On the transverse section, the examined species had cuticle thicknesses varying between 0.19 and 1.15 µm. In 25% of the species, the cuticle appeared smooth. Cuticular striae (Fig. 3a–c) were found in greater abundance in cells of the veins and around trichomes. The epidermises were simple in all taxa. Abaxial (7.48–35.79 µm in height) and adaxial (6.22–40.69 µm in height) epidermises were variable in size. The epidermal cells were generally rectangular in shape and rarely square. In some species, the epidermal cells appeared ovoidal in the periclinal walls due to the elevation of the outer periclinal wall. The outer periclinal wall was often thicker than the internal one, as observed in *Dyssodia papposa*, *Laennecia sophiifolia* and *Schkuhria pinnata*. In some species belonging to the Coreopsideae, Heliantheae and Tageteae tribes, some contents were stained a dark color in the cell lumen of the epidermis. The stomata were located at the same level as other epidermal cells (Fig. 3d) except for some species of the Eupatorieae and Heliantheae tribes, for which they were localized at a higher level than other epidermal ordinary cells (Fig. 3e).

### Mesophyll

**Fig. 4:**
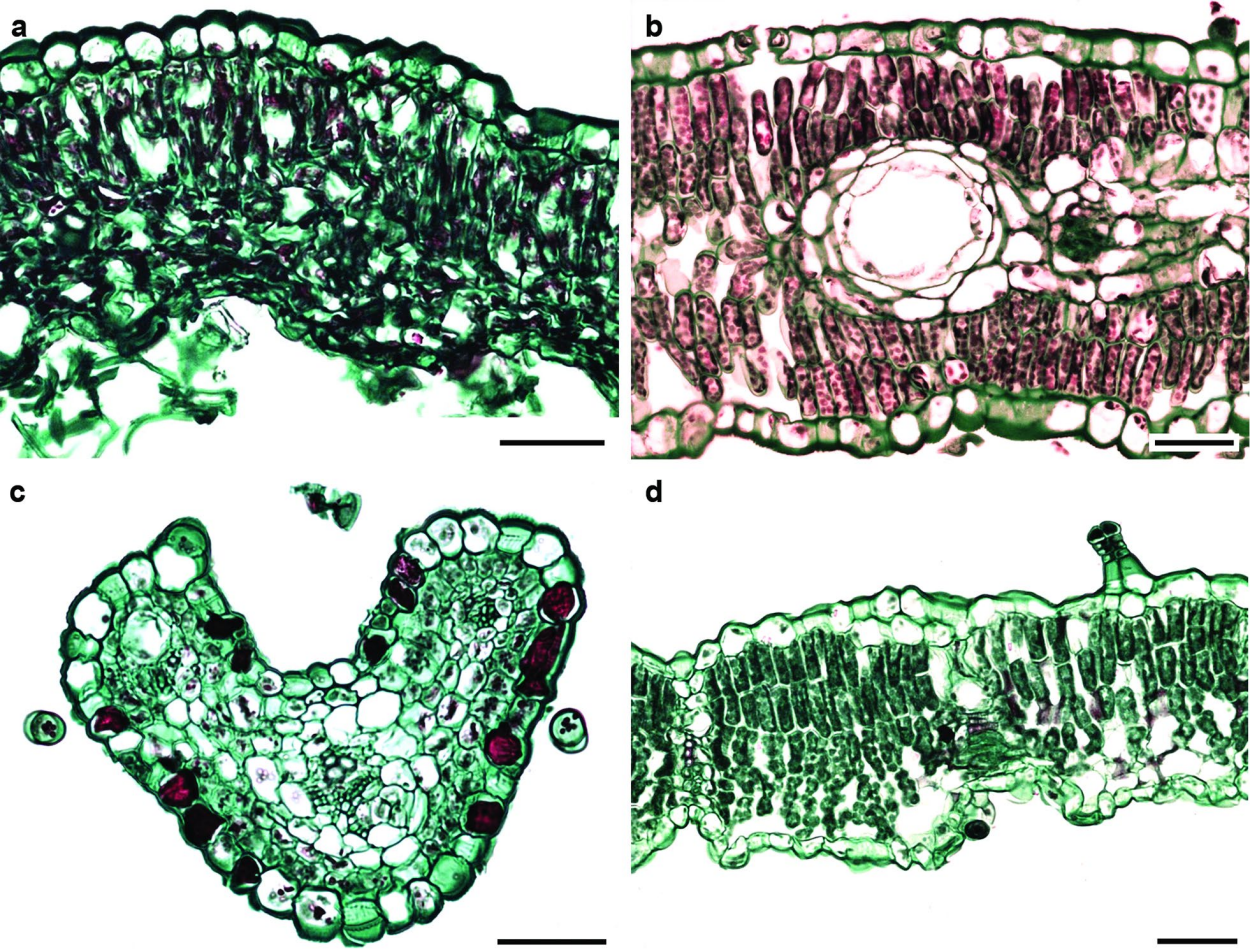
Variation of mesophyll in Asteraceae. **a** *Artemisia ludoviciana* (Tribe Anthemideae). **b** *Baccharis salicifolia* (Astereae). **c** *Cosmos bippinatus* (Coreopsideae). **d** *Brickellia veronicifolia* (Eupatorieae). *Bar* is 20 µm

**Fig. 5:**
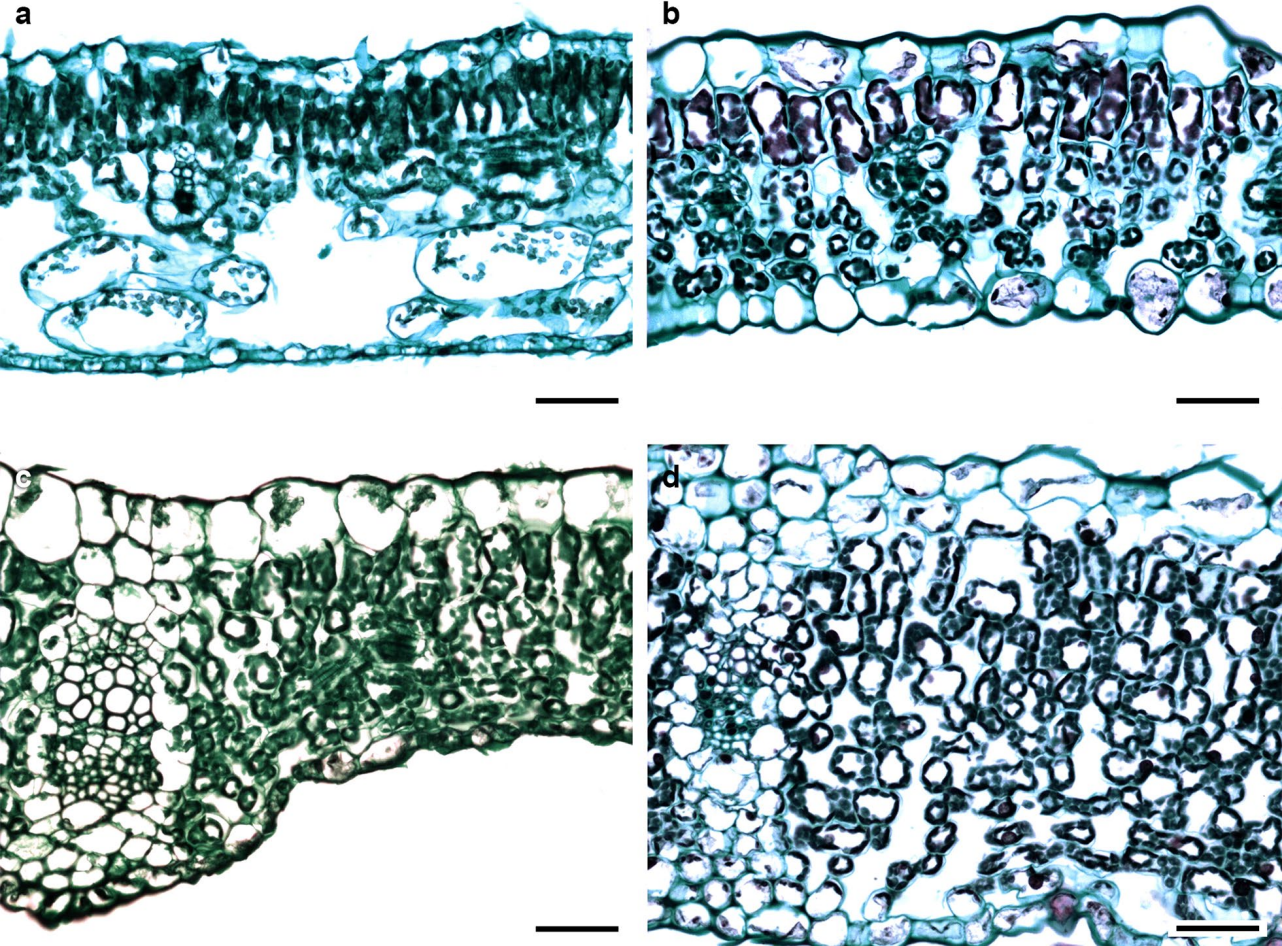
Variation of mesophyll in Asteraceae. **a** *Jaegeria hirta* (Tribe Millerieae). **b** *Montanoa grandiflora* (Heliantheae). **c** *Sonchus oleraceus* (Cichorieae). d. *Pittocaulon praecox* (Senecioneae). Bar is 20 µm

A hypodermis was only observed in *Pectis prostrata* from the Tageteae tribe, where the hypodermis was one cell thick. The mesophyll was dorsiventral in most species and only isofacial in five species (Fig. 4a–d). The palisade parenchyma was formed by one to five cell strata, and, in most cases, it occupied more than half of the total mesophyll thickness. In some species of Astereae and Heliantheae, the palisade parenchyma occupied the most of the mesophyll (Fig. 4). The spongy parenchyma was usually compact in 65% of the species and loose in the rest. In some species from Anthemidae, Eupatorieae, Senecioneae and Tageteae tribes, it constituted the whole mesophyll (Fig. 5). There was an aerenchyma in *Jaegeria hirta* (Millerieae). No mineral contents in the mesophyll cells were observed.

### Vascular bundles

Closed collateral vascular bundles were observed (Figs. 4, 5). In most species, they were surrounded by a sheath of one to three parenchyma layers, which were more conspicuous in species from the Astereae and Tageteae tribes. In the Eupatorieae, Heliantheae, Cichorieae and Senecioneae tribes, sheath extensions towards both surfaces were found (Fig. 5c, d). Secretory canals in some species of Astereae, Eupatorieae and Senecioneae were observed within the sheath (Fig. 4b).

### Midvein

**Fig. 6:**
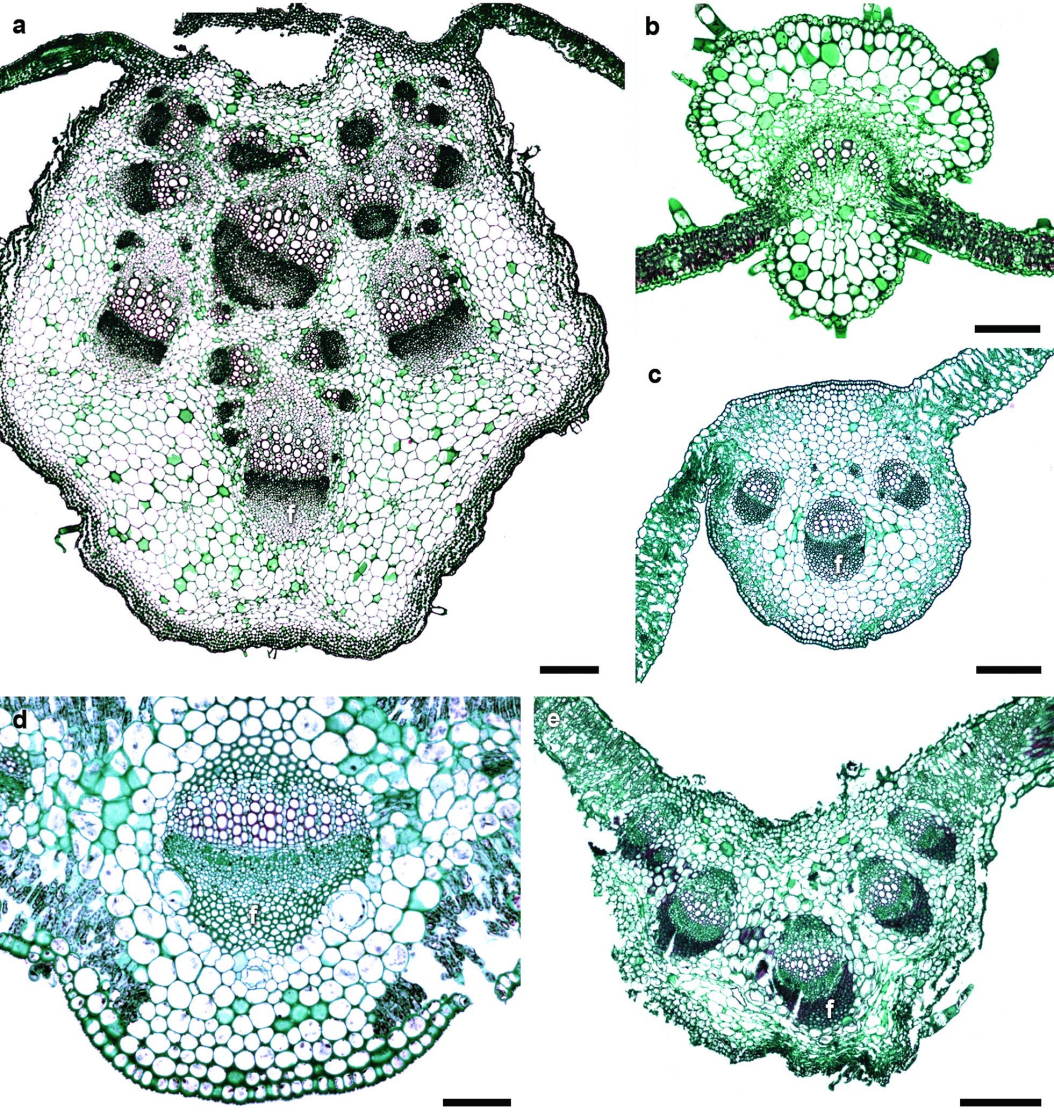
Midvein of some Asteraceae species. a. *Montanoa grandiflora* (Tribe Heliantheae). **b** *Ageratina pichinchensis* (Eupatorieae). **c** *Picris echioides* (Cichorieae). **d** *Baccharis salicifolia* (Astereae). **e** *Acourtia cordata* (Nassauvieae). *Bar* is 300 µm in **a**–**c**, 100 µm in **d**

The midvein occupies the central position of the leaf. The epidermis of the midvein in all species was the same as in the lamina, except for *Acourtia cordata* (Nassauvieae), which had a thicker outer epidermal wall. In this area, the mesophyll and the palisade parenchyma were interrupted by angular collenchyma with 9–16 cell layers. The number of bundles composing the midvein was variable (Fig. 6). The vascular tissue generally formed an arch, with parenchyma at both ends of the vascular bundles, except for *A*. *cordata*, where fibers were observed (Fig. 6a, c–e). No foliar characters were unique to any tribe, and most character states were shared by most tribe members as seen in Table 1.

### Statistical analyses

**Fig. 7:**
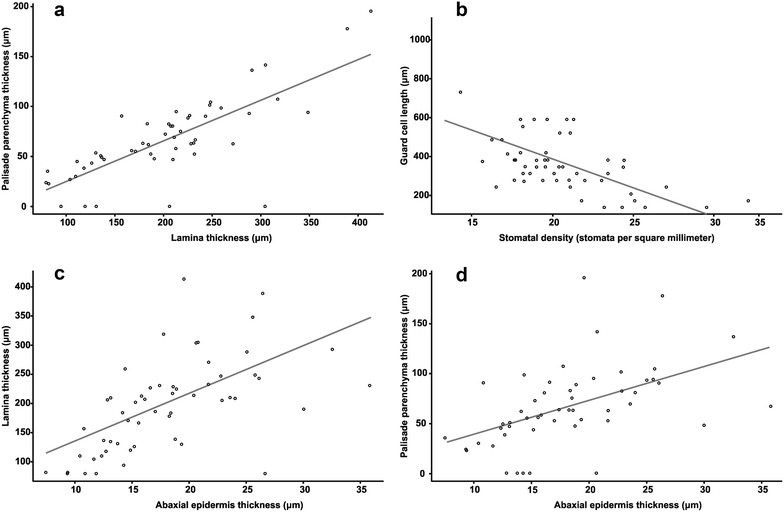
Correlations among pair of anatomical characters. **a** Lamina vs palisade parenchyma thickness. **b** Stomatal density vs guard cell length. **c** Abaxial epidermis vs lamina thickness. **d** Abaxial epidermis vs palisade parenchyma thickness

**Fig. 8:**
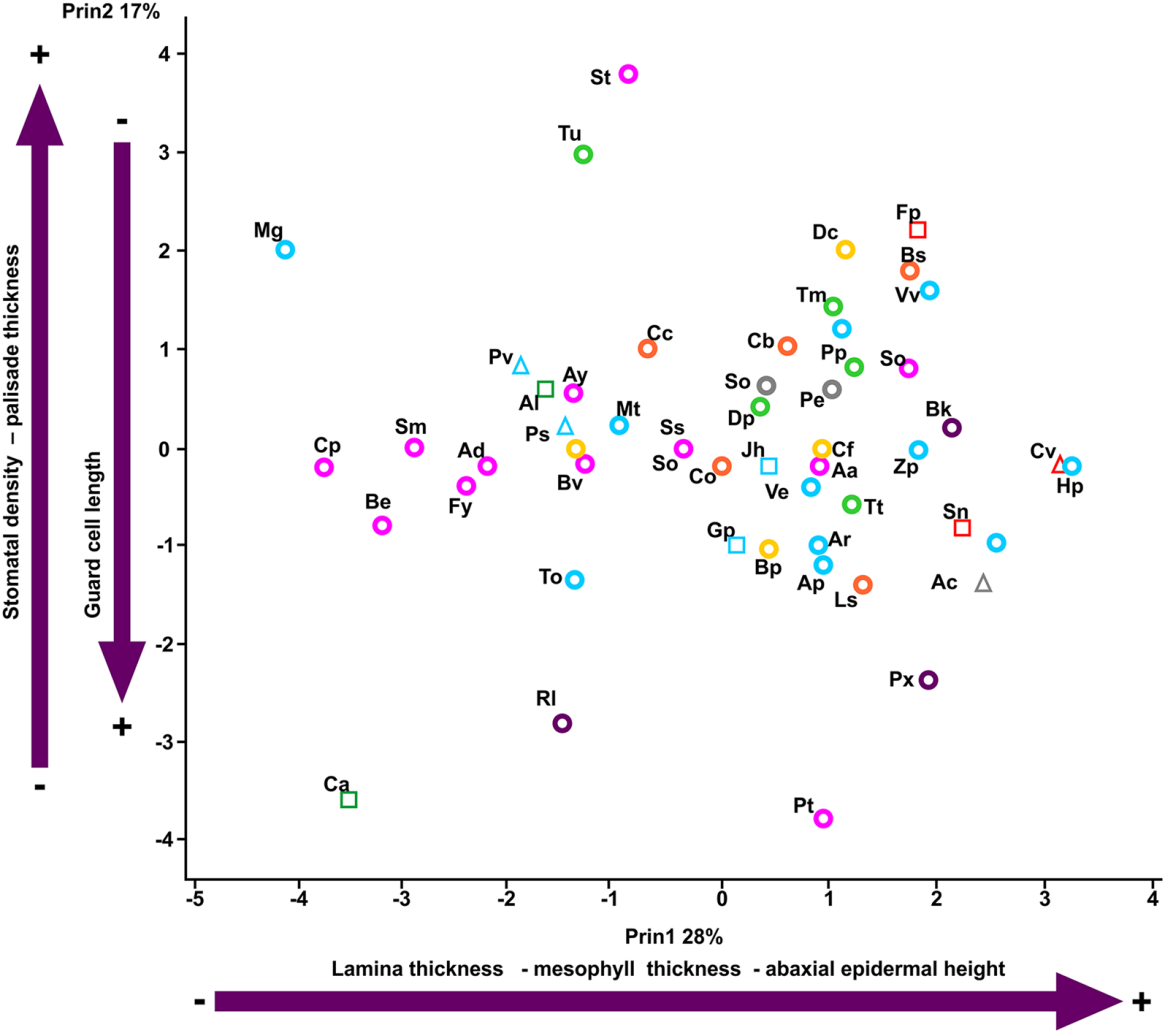
Biplot of the two principal component axis based on leaf characters plotted for species (letters, see information in Additional file 2) and tribes indicated by *colors* and *symbols*

We found significant correlations between some of the studied characters. For example, there is a negative and significant relationship between the guard cell length and stomatal density (Fig. 7). We also found positive significant correlations between leaf thickness and the thickness of palisade and spongy parenchyma (r = 0.66, *P* < 0.001) and between guard cell length and the thickness of the leaves (r = 0.43, *P* = 0.003), other correlations are shown in Fig. 7. PCA explained in four axes 69% of the total variance (Table 2). The first axis explains the leaf thickness whereas the second axis explains the positive values for stomatal density and palisade thickness and negative values for guard cell length (Fig. 8).

**Table 2:** Eigenvector for principal component analysis for the 11 variables in the first four components analyzed for the leaf traits of the 52 species of Asteraceae at the REPSA, Mexico

Characters	Prin 1	Prin 2	Prin 3	Prin 4
Variation explained (%)	28.48	17.11	13.15	10.28
Eigenvalue	3.42	2.05	1.48	1.23
Lamina size	−0.127	0.332	0.455	−0.664
Specific leaf area	−0.216	−0.176	0.029	0.489
Sclerophyll index	−0.197	0.332	0.455	−0.066
Lamina thickness	−0.144	−0.134	0.575	−0.024
Cuticle thickness	−0.016	0.294	−0.058	−0.430
Abaxial epidermis height	0.426	0.059	0.278	0.127
Stomatal density	−0.159	0.518	−0.137	0.214
Guard cell length	0.281	−0.487	0.133	−0.195
Mesophyll thickness	0.510	0.075	0.054	0.142
Palisade thickness	0.216	0.455	0.067	0.204
Spongy thickness	0.138	−0.105	−0.524	0.269

## Discussion

The foliar anatomy of Asteraceae was variable. In the epidermis, differences were found in the shape of epidermal cells, seen in surface view, and in stomata location, as well as epidermis and cuticle thickness. The cell types constituting the mesophyll were variable as well as their abundance. These observations were consistent with the high foliar variation reported for the family (Anderson and Creech 1975; Metcalfe and Chalk 1979; Ferreira et al. 2002; Freire et al. 2005, 2007; Milán et al. 2006; Adedeji and Jewoola 2008). The striae in the leaf cuticle have been previously observed by other authors (Adedeji and Jewoola 2008), who have suggested that the striae could be used to separate species. The observed stomatal types and the stomata being preferably distributed on the abaxial surface agrees with previous reports for the family in other works (Metcalfe and Chalk 1979; Adedeji and Jewoola 2008).

It was difficult to find a defined anatomical pattern for the family. However, we found that the combination of anatomical features is usually more homogenous within the tribes than the family level. For example, most examined species show bifacial leaves, except for some members of the Bahieae, Coreopsidae and Tageteae tribes, which had equifacial leaves. It should also be stressed that all the members of Astereae had amphistomatic bifacial leaves with striated cuticles, while striae were absent in all members of Gnaphalieae. These observations should be confirmed by examining more Astereae and Gnaphalieae members.

### Relationship between the leaves and the habitat

Given that the Asteraceae examined here were found in a habitat with low water availability and poor soils, it was expected that the foliar anatomy would display xerophytic features (Shields 1951; Kramer 1983; Esau 1976; Santos and Ochoa 1990; Fahn and Cutler 1992; Dickison 2000; Cutler et al. 2007). This assertion was not supported by the anatomical features found. The leaves of the Asteraceae studied are better classified as mesomorphic and do not correspond with the vegetation community or the water deficit or poor soils of the volcanic outcrop they leave.

#### Leaf size

It was suggested that a small leaf size is a xeromorphic trait because it is correlated with a reduction in transpiration rate (Fahn and Cutler 1992), so we expected to observe leptophyll, nanophyll or microphyll leaves. However, most of the studied species (46%) have large-sized laminae (mesophyll, macrophyll and rarely megaphyll). Twenty-three percent of the studied species had medium-sized laminae (notophyll), and only 31% of the studied species had small leaves. From the species with larger leaves, 29% had deeply lobed margins, such as *Dahlia coccinea* and *Tagetes tenuifolia*. Half of the species with the larger leaves were ephemeral herbs that only grow during the rainy season, such as *Tithonia tubiformis* (megaphyll) and *Cirsium vulgare* (macrophyll). The other 21% of the species with larger leaves were deciduous that usually respond rapidly to water stress by producing leaves exclusively during the rainy season and losing them when the rain stops, such as *Pittocaulon praecox* (macrophyll) and *Roldana lobata* (macrophyll) that is a perennial herb. Laminar area was expressed as types and this trait was not revealed by our PCA as a variable that contributes to explain the variance in the first four components. The projections of the margin of the lamina (i.e., the lobes and teeth) are interpreted as being related to heat loss morphology (Freire et al. 2005, 2007; Fahn and Cutler 1992; Adedeji and Jewoola 2008; Gil et al. 2012; Schmerler et al. 2012; Redonda-Martínez et al. 2016). Eighty-three percent of the studied species had toothed or lobed margins, which is a common combination in Asteraceae (Bailey and Sinnott 1916; Rojas et al. in review).

#### Palisade parenchyma

Gibson (1996) mentioned that non-succulent xerophytes have thicker leaves than mesophytes because they have well developed multi-layered palisade parenchyma. This agrees with our findings because the palisade parenchyma was well developed in most tribes (Table 1), and in some species as *Baccharis salicifolia* (Astereae) and *Lagascea rigida* (Heliantheae) the mesophyll is composed exclusively of palisade parenchyma. The palisade thickness was within the range (94–355 µm) found in other plant families growing in dry environments (Nevo et al. 2000; Rotondi et al. 2003; Bacelar et al. 2004; Gratani and Varone 2004). Palisade thickness was one of the variables with high loading to explain the variation found in the species studied, this agrees with the results of Tian et al. (2016) who found that leaf thickness is closely related to palisade and spongy thickness. This close relationship appears to be a strategy together with phenology to be efficient during the rainy season that is short.

#### SLA, SI and SPI

Specific leaf area is positive correlated with moisture and nutrient availability (Ackerly et al. 2002) and is mainly determined by leaf density and thickness (Ackerly et al. 2002; Meziane and Shipley 2001). SLA was expected to be low (less than 10 mm^2^/mg, Ackerly 2004) because it enhances the photosynthetic rates and the resistance to cell wall collapse under water-stress (Turner 1994; Ackerly 2004). Most of the studied species (78%) have high values of SLA compared with other species of semi-arid environments and are more similar to values found by other authors in temperate and cold forests (15.4–36.3 mm^2^/mg, Chen et al. 2011; Tian et al. 2016). SLA have been negatively correlated with leaf lifespan (Shipley et al. 2006; Tian et al. 2016) and this relationship have been explained as the result of increased photosynthetic capacity per unit of leaf dry mass in plants with shorter leaf lifespan. Since the most of the studied species by us are herbs, the high SLA found can be explained by this correlation. Also, compared to the tree species studied by Tian et al. (2016) SLA of the studied species was significantly higher. This can be interpreted as evidence of the correlation between growth form and SLA.

The SI was expected to be higher than 0.6 which is indicative of sclerophyllous leaves. Sclerophylls are typically associated to low nutrient concentration (Turner 1994). However in all the studied species the SI values was lower than expected (Additional file 2). Together these two variables indicate that the studied leaves can be considered mallacophylls (Turner 1994) and mesomorphic. The SPI found for the plants in this study was higher than that found for Tian et al. (2016) for some tree species within different ecosystems across China. They discuss that a higher SPI may be an adaptive strategy of leaf stomatal traits that results from higher stomatal conductance and therefore increased photosynthetic capacity which maximize carbon gain during the short growing season (Tian et al. 2016).

#### Cuticle and epidermis

We expected to see thick cuticles and a multiseriate epidermis on the leaf surface because those adaptations minimize transpiration rates. However, cuticle thickness was thin compared to other xerophytic plants, which are 2–22 µm thick (Rotondi et al. 2003; Bacelar et al. 2004; Gratani and Varone 2004). Moreover, the thin cuticles found in the REPSA are similar to those reported for species of *Ambrosia*, *Artemisia* and *Encelia* growing in a desert of North America (Gibson 1996). We also expected to see the leaf surface covered with abundant trichomes, but most of the observed species (97%) were glabrous or had trichomes that were scarcely distributed, except for *Pseudognaphalium*, *Artemisia* and *Conyza* which are herbs growing in open spaces. Most authors have based their taxonomic discussions on trichome morphology (Freire et al. 2005, 2007; Adedeji and Jewoola 2008; Gil et al. 2012; Redonda-Martínez et al. 2016). However in the studied species they were not abundant.

#### Stomata

Fahn and Cutler (1992) have hypothesized that amphistomatic leaves may have evolved in response to increasing aridity during the Tertiary period because they increase leaf conductance to CO_2_. Therefore, amphistomatic leaves favored high maximum leaf conductances and are present in species growing in arid environments (Camargo and Marenco 2011), especially in herbs and shrubs from different environments such as successional forest, deserts or swamps (Mott et al. 1982; Mott and Michaelson 1991). In this study, 60% of the observed species had amphistomatic leaves and agrees with Mott and Michaelson (1991) findings in *Ambrosia cordifolia* because most of these species live in full sun during the short rainy season. The stomata in the studied species were at the same level as other epidermal cells. This pattern is different from the one observed in xeromorphic plants, where stomata are usually sunken or protected in crypts. The mean stomatal density found in this study (362 stomata/mm^2^) was within the range found for other plants growing in arid environments (133–537 stomata/mm^2^. Fahn and Cutler 1992; Bacelar et al. 2004; Yiotis et al. 2006; Gil et al. 2012). However, the stomatal densities observed in this environment (Table 1) are lower but not significantly different than those found in mesic environments (462–846 stomata/mm^2^, Popma et al. 1992; Camargo and Marenco 2011). Wood (1934) suggested that although stomatal density increases with aridity, variations in stomatal density in sclerophyllous forests of South Australia were more related to intra-family characteristics rather than environmental conditions. It is possible that the observed variation in stomatal density has a phylogenetic signal, thus further analysis with a larger Asteraceae sampling growing in different communities is needed to support this assertion. The observed stomatal lengths are similar to the values reported by other authors for Asteraceae growing in arid and temperate environments (16–24 µm, Bacelar et al. 2004; Yiotis et al. 2006; Gil et al. 2012). Both stomatal density and length showed a significant negative scaling as found for other species of different families (Hetherington and Woodward 2003; Pearce et al. 2006; Camargo and Marenco 2011) and were important traits that explained part of the variance according to PCA suggesting that they adjust with palisade thickness and spongy parenchyma to maintain efficient photosynthesis.

#### Dark staining deposits and oils

It has been suggested that the presence of specialized cell types, such as oil containing cells or tannin cells, may be advantageous in dry environments because they protect the mesophyll cells against excess radiation or against herbivores and help to reduce the evaporation rate by interfering with water movement through the leaves (Fahn and Cutler 1992; Jordaan and Theunissen 1992; Turner 1994). Although we did not perform histochemical tests for tannins, the dark staining deposits that we described may be these contents. Thirty percent of the studied species had oil glands associated to vascular bundles, including *B. salicifolia* and *Barkleyanthus salicifolius*. Dark staining deposits were found only in two species (*Cosmos parviflorus* and *D. papposa*).

#### Allometric relationships within the leaf

The correlation between abaxial epidermis thickness and palisade thickness can be viewed from a functional perspective since it can be interpreted as a relationship between the gas exchange mechanism and the photosynthetic tissue resulting in greater efficiency of the leaf (Shields 1951; Rotondi et al. 2003). Our results of the PCA confirms the close allometric relationships between lamina thickness, palisade and spongy parenchyma and abaxial epidermis found by John et al. (2013) in a sample including various angiosperm families. This relationship has been interpreted as evidence of coordinated changes of the tissues composing the leaf (Brodribb et al. 2013). Particularly the correlation between guard cell length and leaf thickness may be showing that the changes in cell size are key to the coordinated variation of leaf size.

### Leaves classification

Some of the studied species showed indicator traits of each of three types of response strategies to water stress and absence of soil. In species of the Eupatorieae (2 spp: *Ageratina adenophora* and *Stevia tomentosa*), Tageteae (3 spp: *D. papposa*, *T. micrantha*, *Pectis prostrata*), and Heliantheae (*L. rigida*) tribes, some foliar features corresponding to the avoidance strategy—water loss minimization—were observed. Those characteristics were hypostomatic or bifacial leaves with well developed, many-layers-thick palisade parenchyma, generally striated cuticles, thick epidermises, and vascular bundles surrounded by a well differentiated parenchyma sheath. In accordance with the foliar anatomy some other plant features indicate that these species prefer the avoidance strategy: Eupatorieae species that are generally shrubs or perennial herbs, there are rhizomes allowing them to store water and all leaves from the examined species of Tageteae were lobulate. The tolerance strategy was observed in all the species from the Senecioneae tribe. They showed traits such as leaf abscission during the higher stress season (November–May), mucilage accumulation (in the shoot), and perennation structures that allow regrowth. Some species from the Astereae tribe (4 spp, including *Conyza canadensis* and *L. sophiifolia*), and some members of Coreopsidae (e.g. *C. parviflorus*) and Heliantheae (e.g. *Tithonia tubiformis*) tribes, showed an escape strategy because they usually developed their life cycle during the rainy season (June–October) and produced seeds before water stress conditions arrived.

Most of the species showed a combination of characters that made their classification difficult. For example, species from the Bahieae and Carduae tribes are annual herbs that grow mainly from June to October; thus, they could be considered escapists. However, these species showed some avoidance features, such as lobulate leaves with striated cuticles and a well-developed palisade parenchyma. This agrees with the asseveration from some authors (Jones 1992; Chaves et al. 2003) about the three strategies not being mutually exclusive but instead we can find a combination of indicator traits of all three strategies within the same plant. The different character combinations for each species could be explained as a result of a fast evolutionary process as proposed by Stebbins (1952) for some members of Cichorieae (Asteraceae).

## Conclusions

The foliar anatomy of Asteraceae was variable, even among members of the same tribe growing in the same locality. If the observed variations were the result of adaptation to a xeric and poor-soil environment, then the observed features would correspond to non-succulent xeromorphic leaves but it was not the case. The majority of the studied species possess mesomorphic leaf features as simple lamina, single-layered epidermis, and soft (mallacophyllous) large-size leaves with high SLA. Although some characters of drought resistance can be observed as for well-developed palisade and parenchyma bundle sheaths. The aforementioned character combination made difficult to classify the species studied within one of the three main response strategies to water stress. We suggest that the occurrence of specific character combinations in each species is due to a very fast evolutionary process involving the growth form, the adaptation to the environment and the phylogeny of the family. A character evolution analysis in an Asteraceae phylogeny including genera endemic to Mexico is needed in order to support this hypothesis (Terrazas, on going research).

The combined study of morphological and anatomical traits and its correlations is important to understand the constraints imposed over the diversity generated by phylogeny and adaptation. For example, although leaf anatomy is not xeromorphic in the studied species, it is advantageous for the species living in the REPSA to have the combination of traits mentioned. Those traits allow them to survive in full sun during the short rainy season having efficient photosynthetic capacity. Assuming that species from the forests around the Xitle colonized the REPSA, it is not surprising that they retained some of their mesic characters and also adapted to the low water environment. There were also species with Neotropical and Nearctic affinities that probably had to modify their leaves to colonize new environments. These species probably retained the capacity to adapt to dry environments, and that is one of the reasons for their success. It is known that in ecological succession, pioneer species need to have low nutritional requirements and efficient metabolisms to survive. The invasiveness of Asteraceae species is probably related to their capacity to grow in this poor-soil environment.

## Additional files

**Additional file 1.** Voucher information for species used in this study. All specimens deposited in Herbario Nacional de México (MEXU), Instituto de Biología, Universidad Nacional Autónoma de México.

**Additional file 2.** Leaf characters by species in Asteraceae.

## Abbreviations

REPSA: Pedregal de San Ángel Ecological Reserve
UNAM: National Autonomous University of Mexico
SLA: specific leaf area
SI: sclerophylly index
SPI: stomatal pore area index
PCA: principal component analysis
