# Supplementary material for: Meso- or xeromorphic? Foliar characters of Asteraceae in a xeric scrub of Mexico
Source: Bot Stud. 2017 Feb 23;58:12. doi: 10.1186/s40529-017-0166-x (PMC5430588; doi:10.1186/s40529-017-0166-x)
Supplement: Supplementary file 1 — Additional file 1. Voucher information for species used in this study. All specimens deposited in Herbario Nacional de México (MEXU), Instituto de Biología, Universidad Nacional Autónoma de México. [file 40529_2017_166_MOESM1_ESM.pdf]

Meso- or xeromorphic? Foliar characters of Asteraceae in a xeric scrub of Mexico

Botanical Studies

Patricia Rivera[1,2], Teresa Terrazas[1], José Luis Villaseñor[1]

[1] Instituto de Biología, Universidad Nacional Autónoma de México, Departamento de Botánica, Apartado Postal 70-367, 04510 Mexico City, Mexico

[2] Posgrado en Ciencias Biológicas, Universidad Nacional Autónoma de México.

*Corresponding author: rivera.perezpatricia@gmail.com

Additional file1. Voucher information for species used in this study. All specimens deposited in Herbario Nacional de México (MEXU), Instituto de Biología, Universidad Nacional Autónoma de México.

Anthemidae: Artemisia ludoviciana Nutt: *O. Hinojosa 443*. Cotula australis (Sieber ex Spreng.) Hook. f: *O. Hinojosa 476*. **Astereae**: Baccharis salicifolia (Ruiz & Pav.) Pers: *O. Hinojosa 438*.Conyza bonariensis (L.) Cronquist: *O. Hinojosa 71, 477*. Conyza canadensis (L.) Cronquist: *O. Hinojosa 740*. Conyza coronopifolia Kunth: *O. Hinojosa 739*. Laennecia sophiifolia (Kunth) G.L. Nesom: *O. Hinojosa 222*. **Bahieae**: Florestina pedata (Cav.) Cass: *L. Céspedes 203*, *O. Hinojosa 470*. Schkuhria pinnata (Lam.) Kuntze ex Thell: *O. Hinojosa 469*. **Cardueae**: Cirsium vulgare (Savi) Ten: *O. Hinojosa 506*. **Cichorieae (Lactuceae)**: Picris echioides L: *O. Hinojosa 478*. Sonchus oleraceus L: *O. Hinojosa 456*. Taraxacum officinale G. H. Weber ex Wigg: *O. Hinojosa 525*. **Coreopsideae (Heliantheae)**: Bidens odorata Cav: *O. Hinojosa 518*. Bidens pilosa L: *O. Hinojosa 503*. Cosmos parviflorus (Jacq.) Pers: *O. Hinojosa 439*. Dahlia coccinea Cav: *O. Hinojosa 446*. Heterosperma pinnatum Cav: *O. Hinojosa 515*. **Eupatorieae**: Ageratina adenophora (Spreng.) R.M. King & H. Rob:*O. Hinojosa 563*. Ageratina cylindrica (McVaugh) R.M. King & H. Rob: *O. Hinojosa 500*. Ageratina deltoidea (Jacq.) R.M. King & H. Rob: *O. Hinojosa 499*. Brickellia secundiflora (Lag.) A. Gray: *O. Hinojosa 555*. Brickellia veronicifolia (Kunth) A. Gray: *O. Hinojosa 497*. Chromolaena pulchella (Kunth) R.M. King & H. Rob: *O. Hinojosa 559*. Fleischmannia pycnocephala (Less.) R.M. King & H. Rob: *O. Hinojosa 554*. Piqueria trinervia Cav: *O. Hinojosa 216*. Stevia micrantha Lag: *O. Hinojosa 527*. Stevia origanoides Kunth: *L. Céspedes 668*, *O. Hinojosa 523*. Stevia salicifolia Cav: *L. Céspedes 638*. *O. Hinojosa 498*. Stevia tomentosa Kunth: *O. Hinojosa 433*. **Gnaphalieae (Inuleae)**: Pseudognaphalium semilanatum (DC.) Anderb: *O. Hinojosa 516*. Pseudognaphalium viscosum (Kunth) Anderb:*O. Hinojosa 504*. **Heliantheae**: Acmella repens (Walter) Rich: *O. Hinojosa 519*. Ambrosia psilostachya DC: *O. Hinojosa 507*. Lagascea rigida (Cav.) Stuessy: *O. Hinojosa 483*. Montanoa grandiflora DC.: *L. Céspedes 607*; *O. Hinojosa 436*. Montanoa tomentosa Cerv: *O. Hinojosa 369*, *F. Soto 593*. Simsia amplexicaulis (Cav.) Pers: *O. Hinojosa 473*. Tithonia tubiformis (Jacq.) Cass: *O. Hinojosa 444*. Verbesina virgata Cav: *O. Hinojosa 437*. Viguiera buddleiiformis (DC.) Benth. et Hook. f. ex Hemsl: *O. Hinojosa 441*. Viguiera excelsa (Willd.) Benth. et Hook. f: *O. Hinojosa 370*. Zinnia peruviana (L.) L: *O. Hinojosa 508*. **Millerieae (Heliantheae)**: Galinsoga parviflora Cav: *O. Hinojosa 475*. Jaegeria hirta (Lag.) Less : *O. Hinojosa 372*; *F. Soto 176*. **Nassauvieae (Mutisieae)**: Acourtia cordata (Cerv.) B. L. Turner: *O. Hinojosa 442*. **Senecioneae**: Barkleyanthus salicifolius (Kunth) H. Rob. & Brettell: *F. Soto 85*. Pittocaulon praecox (Cav.) H. Rob. & Brettell: *O. Hinojosa 505*. Roldana lobata La Llave: *O. Hinojosa 448*. **Tageteae**: Dyssodia papposa (Vent.) A.S. Hitchc: *L. Céspedes 206*, *O. Hinojosa 471.* Pectis prostrata Cav: *O. Hinojosa 524*. Tagetes micrantha Cav: *O. Hinojosa 434*. Tagetes tenuifolia Cav: *O. Hinojosa 474.*
